# Supplementary material for: Characterization of peptide-protein relationships in protein ambiguity groups via bipartite graphs
Source: PLoS One. 2022 Oct 21;17(10):e0276401. doi: 10.1371/journal.pone.0276401 (PMC9586388; doi:10.1371/journal.pone.0276401)
Supplement: S1 Table — (PDF) [file pone.0276401.s001.pdf]

S1 Table: Technical information and search engine parameters for the three analyzed data quantitative proteomics data sets.

Parameter	D1	D2	D3
Mass spectrometer	Q Exactive HF (Thermo Fisher Scientific)	LTQ Orbitrap Velos (Thermo Fisher Scientific)	LTQ Orbitrap (Thermo Fisher Scientific)
Data acquisition type	data-dependent acquisition	data-dependent acquisition	data-dependent acquisition
Software	KNIME workflow with PIA	MaxQuant 1.6.17.0	MaxQuant 1.6.17.0
Search engines	Mascot 2.7<br>MS-GF+<br>X!Tandem	Andromeda	Andromeda
Enzyme	Trypsin	Trypsin	Trypsin, LysC
Max. missed cleavages	2	2	2
Decoy generation	Randomize (concatenated target-decoy-db)	Revert	Randomize
Precursor tol.	5 ppm	20 ppm (first search)<br>6 ppm (main search)	20 ppm (first search)<br>4.5 ppm (main search)
Fragment tol.	20 mmu	0.5 Da	0.5 Da
Fixed PTMs	Carbamidomethyl (C)	Carbamidomethyl (C)	Carbamidomethyl (C)
Variable PTMs	Oxidation (M)<br>Gln-> pyro-Glu (N-terminal Q)<br>Deamidated (NQ)<br>Ammonium (DE)<br>Ammonialoss (N, N-terminal C)	Oxidation (M)<br>Acetyl (Protein N-term)	Oxidation (M)<br>Acetyl (Protein N-term)
