# Supplementary material for: Characterization of peptide-protein relationships in protein ambiguity groups via bipartite graphs
Source: PLoS One. 2022 Oct 21;17(10):e0276401. doi: 10.1371/journal.pone.0276401 (PMC9586388; doi:10.1371/journal.pone.0276401)
Supplement: S2 Table — (PDF) [file pone.0276401.s002.pdf]

S2 Table: Overview over the quantitative peptide-level data sets.

	D1_quant	D2_quant	D3_quant	D3_iso_quant
experimental groups	5	9	2	2
pairwise comparisons	10	36	1	1
quantified peptides*	22,939	8,101	46,544	44,843
mean number of peptides with valid ratio**	18,093	5,619	30,369	29,490

* In at least one sample. Only peptides inside the chosen length range are considered.

** Mean over the pairwise group comparisons.
