# Supplementary material for: Characterization of peptide-protein relationships in protein ambiguity groups via bipartite graphs
Source: PLoS One. 2022 Oct 21;17(10):e0276401. doi: 10.1371/journal.pone.0276401 (PMC9586388; doi:10.1371/journal.pone.0276401)
Supplement: S3 Table — (PDF) [file pone.0276401.s003.pdf]

S3 Table: Influence of different minimal peptide lengths on the bipartite graphs for D2_fasta.

	min 5 AA	min 6 AA	min 7 AA	min 9 AA
protein accessions	6,336	6,335	6,333	6,333
protein nodes	6,265	6,264	6,263	6,263
peptide sequences	752,532	718,476	679,995	603,418
peptide nodes	12,962	8,132	7,563	7,366
edges	26,792	15,029	13,653	12,677
graphs	1,649	4,908	5,471	5,604
graphs with 1 protein node	1,506	4,296	5,045	5,264
isomorphism classes	18	64	41	36
**largest graph***				
protein nodes	4,413	116	69	69
peptide nodes	10,907	356	264	253
edges	24,434	1,292	3,329	3,033
**second largest graph***				
protein nodes	21	70	52	50
peptide nodes	37	267	203	181
edges	131	3,359	743	613

* In terms of number of protein nodes.
