# Supplementary material for: Characterization of peptide-protein relationships in protein ambiguity groups via bipartite graphs
Source: PLoS One. 2022 Oct 21;17(10):e0276401. doi: 10.1371/journal.pone.0276401 (PMC9586388; doi:10.1371/journal.pone.0276401)
Supplement: S4 Table — (PDF) [file pone.0276401.s004.pdf]

S4 Table: Influence of different minimal peptide lengths on the bipartite graphs for D3_fasta (without isoforms).

	min 5 AA	min 6 AA	min 7 AA	min 9 AA
protein accessions	81,591	81,572	81,548	81,440
protein nodes	80,932	80,897	80,856	80,676
peptide sequences	3,309,331	3,204,104	3,050,340	2,733,226
peptide nodes	192,162	157,556	148,555	143,264
edges	699,493	480,830	431,391	401,884
graphs	4,576	14,177	20,270	22,327
graphs with 1 protein node	3,722	8,178	10,129	11,088
isomorphism classes	253	2,305	4,198	4,522
**largest graph***				
protein nodes	74,157	40,266	6,472	2,203
peptide nodes	183,383	89,697	14,993	5,106
edges	685,438	315,173	56,950	17,897
**second largest graph***				
protein nodes	27	86	306	229
peptide nodes	51	126	757	454
edges	198	1,412	2,884	2,084

* In terms of number of protein nodes.
