# Supplementary material for: Characterization of peptide-protein relationships in protein ambiguity groups via bipartite graphs
Source: PLoS One. 2022 Oct 21;17(10):e0276401. doi: 10.1371/journal.pone.0276401 (PMC9586388; doi:10.1371/journal.pone.0276401)
Supplement: S5 Table — (PDF) [file pone.0276401.s005.pdf]

S5 Table: Comparison of bipartite graph characteristics without and with isoforms on data set D3 (with minimal peptide length of seven amino acids).

	D3_fasta	D3_iso_fasta	D3_quant	D3_iso_quant
protein accessions	81,548	103,541	17,585	22,216
protein nodes	80,856	102,463	10,969	11,672
peptide sequences	3,050,340	3,154,386	30,369	29,490
peptide nodes	148,555	184,932	10,540	10,895
edges	431,391	647,037	23,802	27,038
graphs	20,270	20,048	5,267	5,162
graphs with 1 protein node	10,129	8,948	3,315	3,106
isomorphism classes	4,198	5,657	459	524
**largest graph***				
protein nodes	6,472	8,895	57	58
peptide nodes	14,993	19,123	65	67
edges	56,950	87,852	350	359
**second largest graph***				
protein nodes	306	434	35	37
peptide nodes	757	940	34	34
edges	2,884	4,613	181	185

* In terms of number of protein nodes.
