# Supplementary material for: Characterization of peptide-protein relationships in protein ambiguity groups via bipartite graphs
Source: PLoS One. 2022 Oct 21;17(10):e0276401. doi: 10.1371/journal.pone.0276401 (PMC9586388; doi:10.1371/journal.pone.0276401)
Supplement: S1 Fig — The peptide length is given in amino acids. As an example, here the values for data set D1 are shown. Uniqueness is here defined as belonging to only one protein node, which may consist of multiple protein accessions. (PDF) [file pone.0276401.s006.pdf]

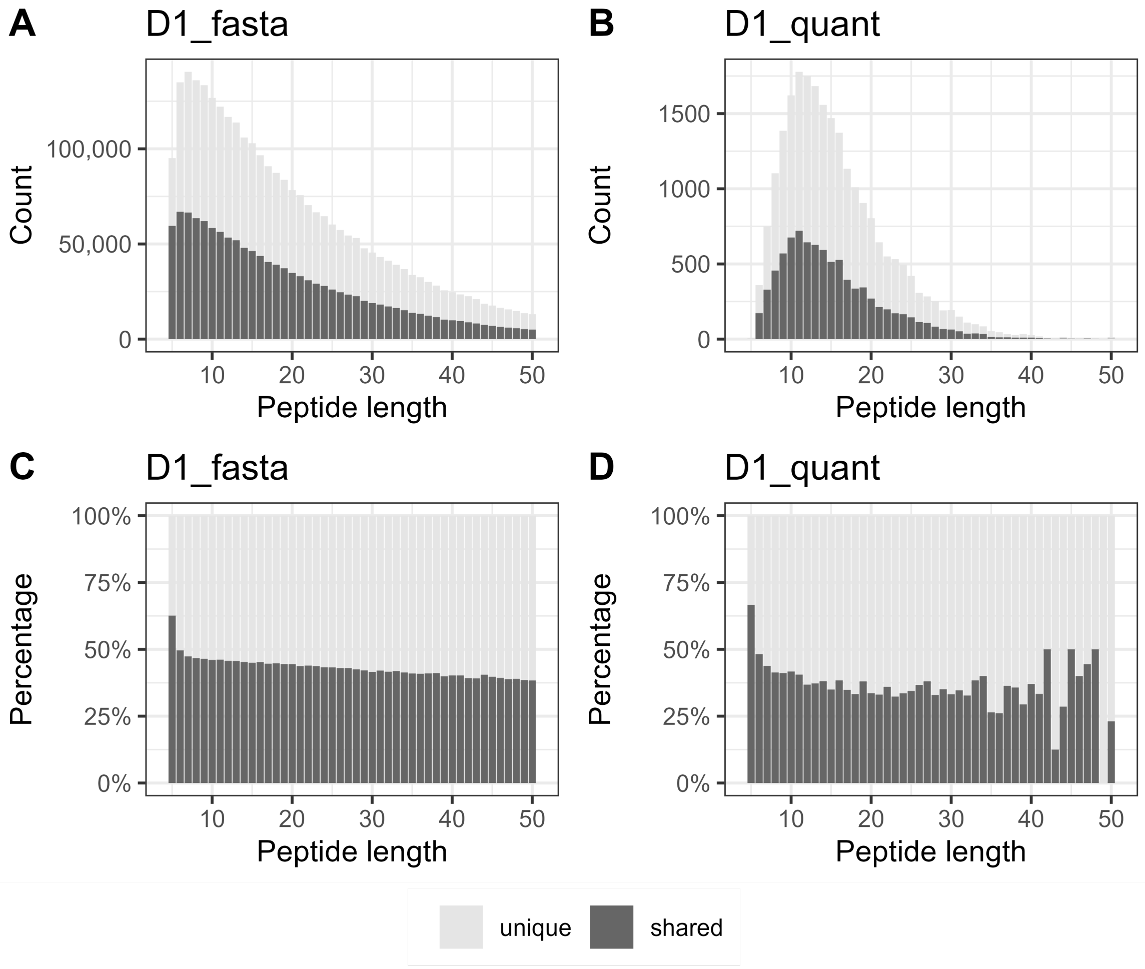

S1 Figure: Count and percentages of shared and unique peptide sequences depending on the peptide length. The peptide length is given in amino acids. As an example, here the values for data set D1 are shown. Uniqueness is here defined as belonging to only one protein node, which may consist of multiple protein accessions.
