# Supplementary material for: Characterization of peptide-protein relationships in protein ambiguity groups via bipartite graphs
Source: PLoS One. 2022 Oct 21;17(10):e0276401. doi: 10.1371/journal.pone.0276401 (PMC9586388; doi:10.1371/journal.pone.0276401)
Supplement: S2 Fig — The leftmost column shows the distribution of number of protein nodes for D1, D2 and D3, on database level (*_fasta) and on the level of quantified peptides (*_quant). On the x-axis the number of protein nodes are shown and on the y-axis the proportion of graphs with this exact number of protein nodes in comparison to all graphs. E.g., the bar at x = 1 is the proportion of graphs with only one protein node compared to all graphs. The sum of all bar heights adds up to one for each subfigure. Similarly, the distribution of the number of total peptide nodes, unique peptide nodes and shared peptide nodes are shown in the following columns. Minimal peptide lengths were chosen as seven for D1 and D3 and six for D2. The rightmost bar with the label “10+” comprises the values of 10 and above. (PDF) [file pone.0276401.s007.pdf]

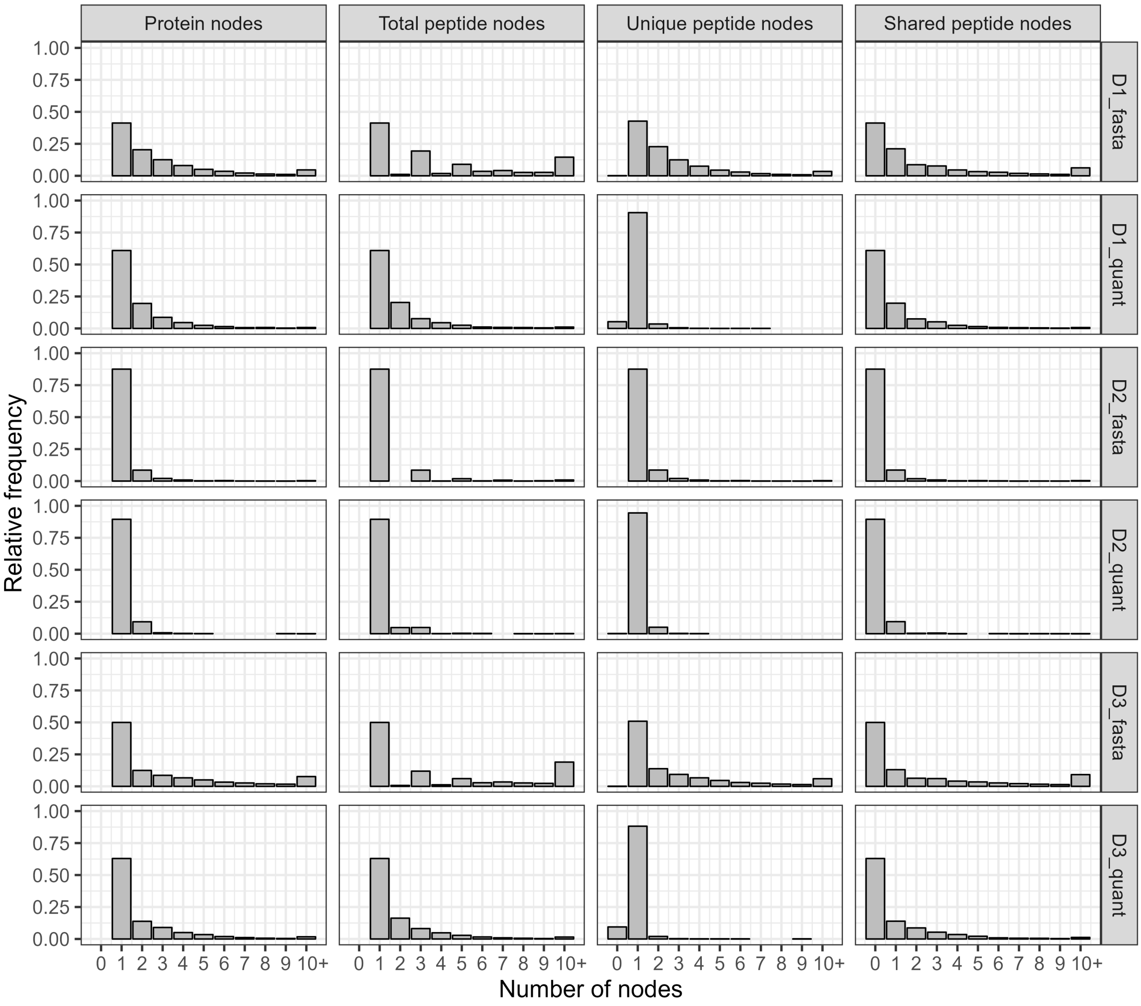

S2 Figure: Distribution of numbers of different node types for the bipartite peptide-protein graphs. The leftmost column shows the distribution of number of protein nodes for D1, D2 and D3, on database level (*_fasta) and on the level of quantified peptides (*_quant). On the x-axis the number of protein nodes are shown and on the y-axis the proportion of graphs with this exact number of protein nodes in comparison to all graphs. E.g., the bar at x = 1 is the percentage of graphs with only one protein node compared to all graphs. The sum of all bar heights adds up to one for each subfigure. Similarly, the distribution of the number of total peptide nodes, unique peptide nodes and shared peptide nodes are shown in the following columns. Minimal peptide lengths were chosen as seven for D1 and D3 and six for D2. The rightmost bar with the label ”10+” comprises the values of 10 and above.
