# Supplementary material for: Characterization of peptide-protein relationships in protein ambiguity groups via bipartite graphs
Source: PLoS One. 2022 Oct 21;17(10):e0276401. doi: 10.1371/journal.pone.0276401 (PMC9586388; doi:10.1371/journal.pone.0276401)
Supplement: S4 Fig — (a) D3_fasta, (b) D3_quant, with number of occurrences and percentage of all graphs. (PDF) [file pone.0276401.s009.pdf]

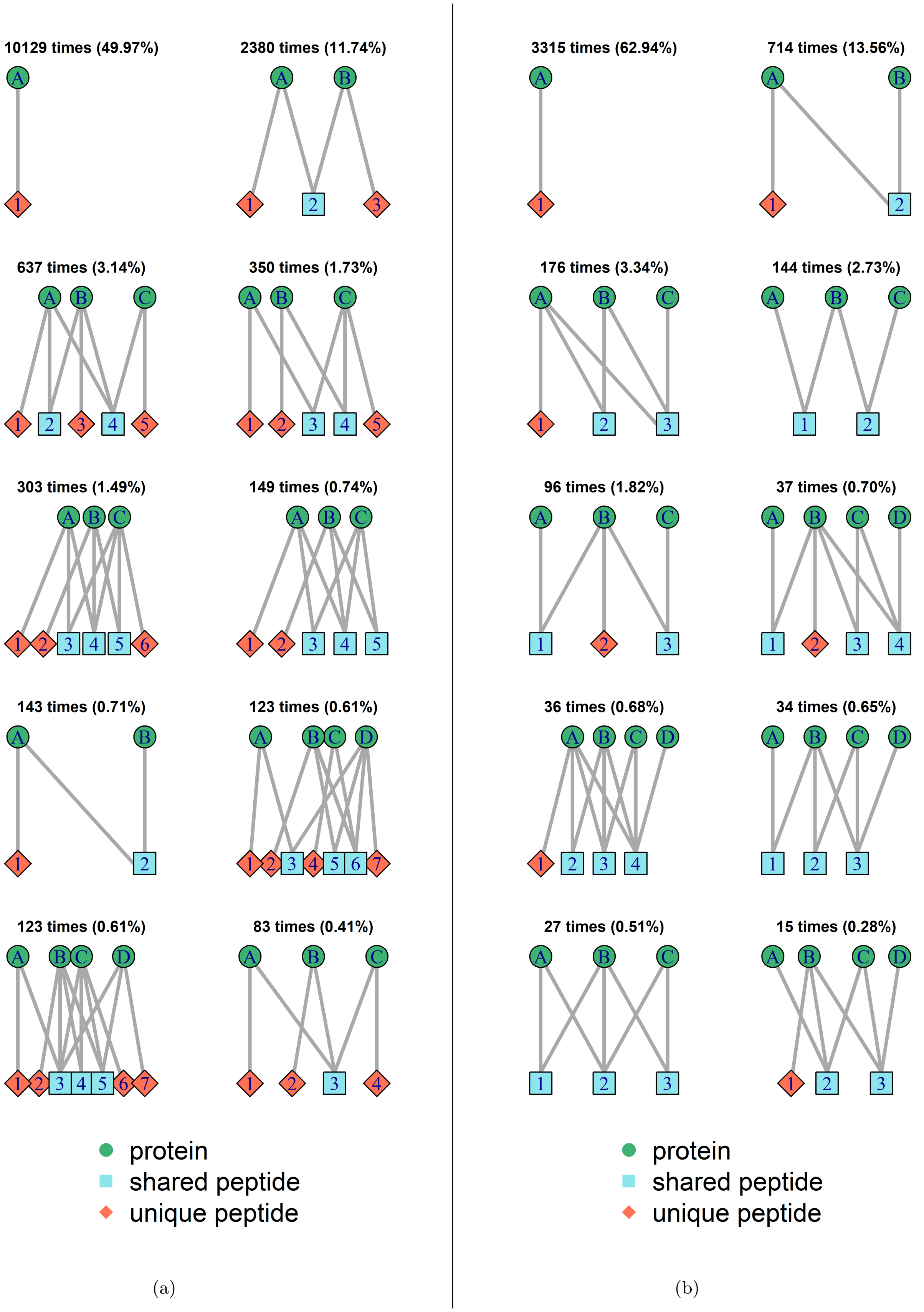

(a) (b)

S4 Figure: Representative bipartite graphs of the ten largest isomorphism classes found in data set D3 (a) D3_fasta, (b) D3_quant, with number of occurrences and percentage of all graphs.
